# *Hanceola suffruticosa* (Lamiaceae, Nepetoideae), a new species from the Sino-Vietnamese border

**DOI:** 10.3897/phytokeys.145.49995

**Published:** 2020-04-10

**Authors:** Ya-Ping Chen, Alan J. Paton, Chun-Lei Xiang

**Affiliations:** 1 CAS Key Laboratory for Plant Diversity and Biogeography of East Asia, Kunming Institute of Botany, Chinese Academy of Sciences, Kunming, 650201, China Kunming Institute of Botany, Chinese Academy of Sciences Kunming China; 2 Royal Botanic Gardens, Kew, Richmond, TW9 3AB, UK Royal Botanic Gardens London United Kingdom

**Keywords:** *
Hanceola
*, Hanceolinae, new species, Ocimeae, Sino-Vietnamese border

## Abstract

*Hanceola* is a genus of eight herbaceous species previously thought to be endemic to southern China. However, *H. suffruticosa*, a new species described here from China and Vietnam, differs from all other species of *Hanceola* by its subshrubby habit. It is also distinct in its shallowly bicrenate laminae and densely purplish glandular puberulent inflorescences. The morphological description, illustrations, and distribution of the new species are presented. A key to all species of *Hanceola* is also provided.

## Introduction

Comprising about eight species, the genus *Hanceola* Kudô (Ocimeae, Nepetoideae, Lamiaceae) is endemic to the evergreen and mixed forests in southern China (Wu and Li 1977; Li and Hedge 1994; Harley et al. 2004). It can be distinguished from other genera of Ocimeae based on the following set of characters: cymes pedunculate and bracteolate, calyx 2-lipped (3/2, a 3-toothed posterior lip with median tooth larger, and a 2-toothed anterior lip), corolla 2-lipped (2/3, 2 lobes on the posterior lip and 3 lobes on the anterior) with tube clearly dilating at midpoint, and free filaments inserted near the throat of corolla (Wu and Li 1977; Li and Hedge 1994; Paton and Ryding 1998; Harley et al. 2004).

Hemsley established the genus *Hancea* Hemsl. with type species *H. sinensis* Hemsl. based on two syntypes, Faber 666 and 681, both collected from Mt. Omei of Sichuan Province (Forbes and Hemsley 1890). Considering that *Hancea* Hemsl. was a late homonym of *Hancea* Seem. (Euphorbiaceae), Kudô (1929) proposed a new name *Hanceola* Kudô for *Hancea* Hemsl.. Tribe Hanceoleae was proposed by Wu and Li (1977) in subfamily Ocimoideae sensu Briquet (1895–1897) to accommodate the distinct genus. Based on morphological cladistic analysis, Cantino (1992a, b) assigned all taxa of Ocimoideae to Nepetoideae sensu Cantino et al. (1992) and recognized Ocimoideae as tribe Ocimeae of subfamily Nepetoideae. Cantino et al. (1992) also placed the genus *Siphocranion* Kudô as a synonym under *Hanceola*. Since *Siphocranion* is distinct from *Hanceola* in its sessile and single-flowered cymes, Paton and Ryding (1998) resurrected *Siphocranion* from *Hanceola*, and treated the two genera as *incertae sedis* in Ocimeae together with *Isodon* (Schrad. ex Benth.) Spach. Harley et al. (2003) later established the subtribe Hanceolinae in Ocimeae to accommodate the three genera, which is adopted in the recent classification of Lamiaceae (Harley et al. 2004).

Zhong et al. (2010) first elucidated the phylogenetic relationships within Ocimeae based on molecular phylogenetic analyses. Using the nuclear ribosomal internal transcribed spacer (nrITS) and two plastid DNA regions (*rps16* and *trnL-trnF*), they demonstrated that each of the three genera *Siphocranion*, *Hanceola*, and *Isodon* formed a distinct lineage within Ocimeae; the subtribes Siphocranioninae and Isodoninae were thus described to accommodate *Siphocranion* and *Isodon*, respectively (Zhong et al. 2010), while subtribe Hanceolinae is restricted to include *Hanceola* alone. Their results and treatment were further supported by Chen et al. (2019).

During our recent field investigations in Malipo County of Yunnan Province, southwestern China, an unusual species of *Hanceola* was discovered at the Sino-Vietnamese border. Further morphological studies suggested that it represents an undescribed species. Hereafter, we describe it as *Hanceola suffruticosa* Y.P. Chen, A.J. Paton & C.L. Xiang.

## Material and methods

This study was based on comparison of herbarium specimens of *Hanceola* from 13 public herbaria AU, E, FJSI, HHBG, IBK, IBSC, LBG, K, KUN, NAS, PE, SM, and SZ (herbarium acronyms follow Index Herbariorum; Thiers 2020) and our new collections in China (herbarium specimens kept in KUN). Meanwhile, protologues of all published names and other related taxonomic literature (Forbes and Hemsley 1890; Kudô 1929; Sun 1942; Wu and Li 1977; Li and Hedge 1994; Paton and Ryding 1998; Harley et al. 2004) were collated and reviewed. Terminology for the description of the new species followed that of Li and Hedge (1994) and Harley et al. (2004).

## Taxonomic treatment

### Hanceola suffruticosa

Taxon classificationPlantaeLamialesLamiaceae

Y.P. Chen, A.J. Paton & C.L. Xiang sp. nov.

F44BD0E5-153D-5E51-BFFA-B4A3DA29270A

urn:lsid:ipni.org:names:77209337-1

Figs 1 , 2

#### Type.

China. Yunnan Province: Malipo County, Tianbao Town, Bajiaoping Village, under the evergreen mixed forest, elevation 1140 m, 1 Dec 2018, Y.P. Chen & L.Q. Jiang EM748 (holotype: KUN!; isotypes: K!, KUN!, PE!).

#### Diagnosis.

*Hanceola suffruticosa* differs from other species of *Hanceola* by being a subshrub with woody rather than herbaceous stems, shallowly bicrenate margin of laminae rather than coarsely dentate, and densely purplish glandular puberulent inflorescences rather than subglabrous or with white glandular or eglandular hairs.

Subshrubs 50–100 cm tall. Stems woody, erect, branched, old stems greyish brown, subterete, glabrous, young stems and branchlets obtusely 4-angled, densely purplish puberulent. Leaves opposite; laminae oblong, ovate to ovate-lanceolate, papery, 7–22 × 3–9 cm, apex acute to acuminate, base cuneate, margin shallowly bicrenate, adaxially green, subglabrous to sparsely puberulent, abaxially light green or purple, subglabrous, glandular, puberulent on veins; lateral veins 4–5 paired; petioles 2–8 cm long, densely purplish puberulent. Inflorescence axillary and terminal, to 20 cm long, cymes (1–) 3–7-flowered; peduncles 2–5 mm long, pedicels 5–10 mm long, densely purplish glandular puberulent; bracts lanceolate to linear, 2–5 mm long, bracteoles linear, 1–2 mm long, densely purplish puberulent. Calyx campanulate, ca. 4 mm long, 10-veined, densely purplish glandular puberulent and glandular outside, glandular puberulent inside; 2-lipped to 1/2 its length, posterior lip 3-toothed, teeth broadly triangular, apex acuminate, medium lobe larger, anterior lip 2-toothed, teeth triangular, apex acuminate, fruiting calyx dilated to ca. 1.2 cm long, slightly curved. Corolla light purple to reddish purple, 3–4 cm long, sparsely puberulent outside, tube slightly curved, long exserted, ca. 1–2 mm in diameter at base, gradually dilated to 1 cm at apex, sparsely pubescent inside at base; limb 2-lipped, dotted with purple spots inside, posterior lip 2-lobed, lobes reflexed, orbicular, ca. 3 mm long, anterior lip 3-lobed, lobes oblong, ca. 6 mm long. Stamens 4, inserted at apical 1/3 the length of corolla, unequal, anterior pair longer, slightly exserted, posterior pair included; anthers ovoid, cells 2, divaricate, confluent at apex; filaments puberulent. Style included, apex shallowly 2-cleft; disc 4-lobed, lobes alternating with mericarps, anterior lobe much larger. Mericarps yellowish brown, oblong, ca. 3 × 2 mm, glabrous.

**Figure 1.**
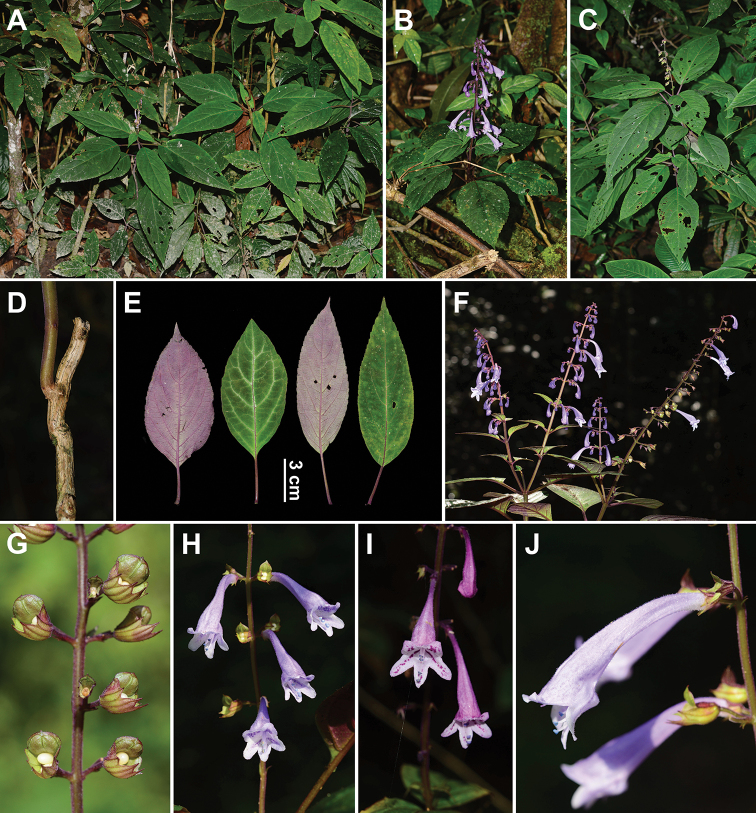
*Hanceola suffruticosa*. **A** Habitat **B, C** plants **D** stem **E** leaves **F** inflorescences **G** post-flowering calyces **H, I** flowers in frontal view **J** flowers in lateral view (Photographs by Ya-Ping Chen).

**Figure 2.**
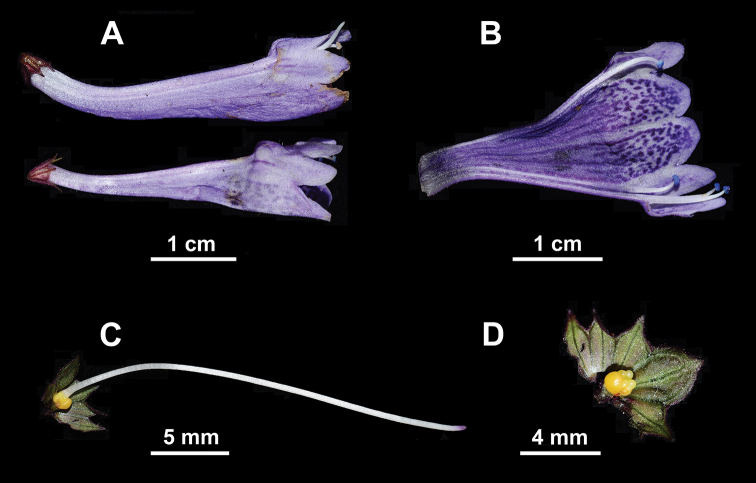
*Hanceola suffruticosa*. **A** Flowers **B** dissected corolla **C** pistil **D** dissected calyx and ovary (Photographs by Ya-Ping Chen).

#### Phenology.

Flowering from October to December, fruiting from November to January next year.

#### Etymology.

The epithet of the new species refers to its suffrutescent habit, which is distinct in the genus.

#### Common name (assigned here).

Mu Jing Si Lun Xiang (木茎四轮香; Chinese name).

#### Distribution and habitat.

**Figure 3.**
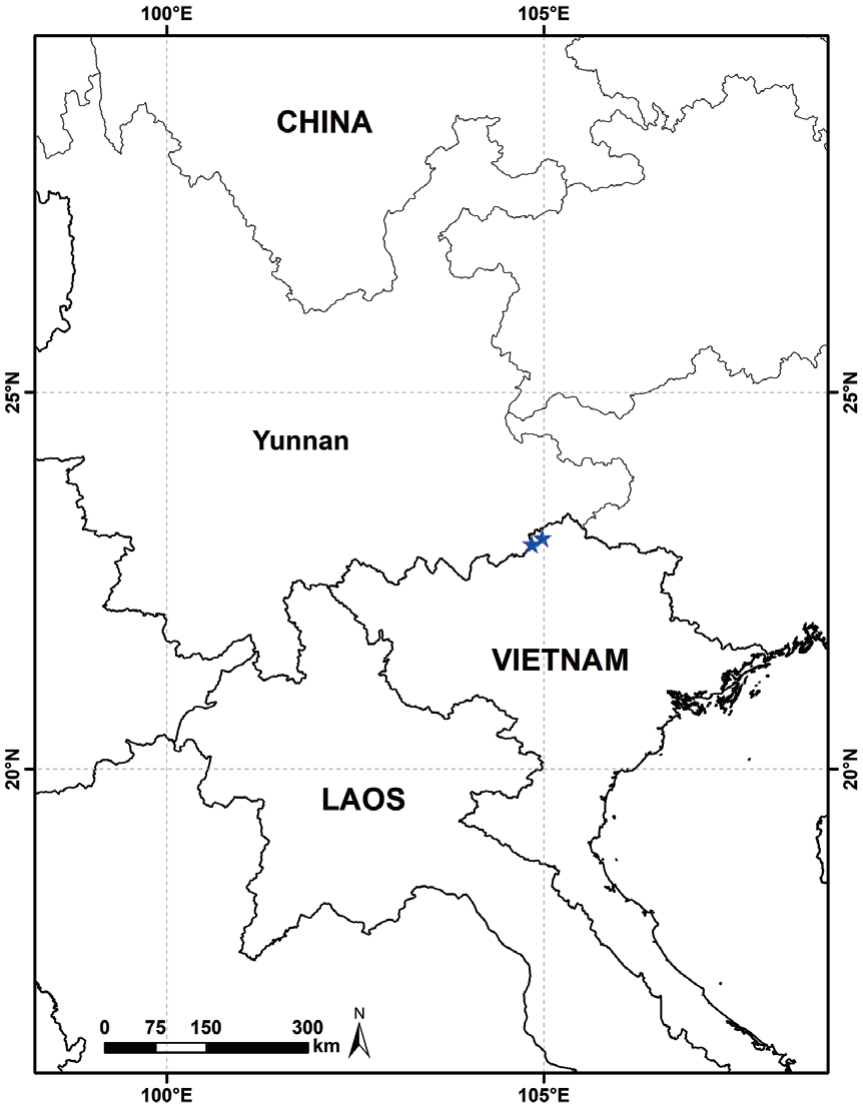
Distribution of *Hanceola suffruticosa* (stars).

*Hanceola suffruticosa* is now only known at the Sino-Vietnamese border of Malipo County in Yunnan Province, China and Quan Ba District of Ha Giang Province, Vietnam (Fig. 3). It grows in the evergreen mixed forests at an elevation of 1100–1150 m.

#### Additional specimens examined.

Vietnam. Ha Giang Province: Quan Ba District, Can Ti Municipality, vicinities of Sing Xuoi Ho Village, in closed evergreen mixed wet forest along tops of karst remnant limestone ridge at elevation 1100–1150 m, 12 Oct 1999, N.T. Hiep et al. NTH3572 (K000479731!).

## Discussion

*Hanceola* can be distinguished from all other genera of tribe Ocimeae by its pedunculate and bracteolate cymes, 2-lipped (3/2) calyx, 2-lipped (2/3) corolla abruptly dilating toward apex, as well as free filaments inserted near the throat of corolla (Wu and Li 1977; Li and Hedge 1994; Paton and Ryding 1998; Harley et al. 2004). Characterized by all these features, the new species we found at the Sino-Vietnamese border is shown to be a member of *Hanceola*. However, unlike other species of *Hanceola*, which are all perennial herbs, *H. suffruticosa* are subshrubs with woody and robust stems. In addition, the new species is distinct in its morphology of laminae and indumentum of branchlets and inflorescences. Specifically, laminae of most *Hanceola* species are lanceolate with base decurrent on petiole and margin coarsely dentate, whereas that of *H. suffruticosa* are oblong, ovate to ovate-lanceolate, with base not decurrent on petiole and margin shallowly bicrenate. Moreover, *H. suffruticosa* are purplish puberulent all over the branchlets and purplish glandular puberulent all over the inflorescences, while branchlets and inflorescences of other species of *Hanceola* are either subglabrous or with whitish glandular or eglandular hairs.

With all other species of *Hanceola* being endemic to southern China, *H. suffruticosa* is now the only species of the genera to be reported from China and Vietnam (Fig. 3). However, consistent with the habitat of other species, *H. suffruticosa* is also accustomed to the shady and moist evergreen mixed forests.

Though only nine species have been reported from *Hanceola*, most of them are only known from several old specimens. More efforts are needed to further reveal the relationships within these species. Here we provide a key to the nine species of *Hanceola* below.

### Key to the species of *Hanceola*

**Table:** 

1	Base of lamina not decurrent on petiole	**2**
–	Base of lamina decurrent on petiole	**3**
2	Subshrubs, corolla slightly curved	***H. suffruticosa***
–	Perennial herbs, corolla strongly curved	***H. cordiovata***
3	Stamens exserted	***H. exserta***
–	Stamens included	**4**
4	Stamens subequal, corolla yellow	***H. sinensis***
–	Anterior stamens longer, corolla purple	**5**
5	Corolla longer than 3 cm	**6**
–	Corolla shorter than 2.5 cm	**7**
6	Lamina elliptic, inflorescence glandular puberulent	***H. tuberifera***
–	Lamina lanceolate, inflorescence subglabrous	***H. labordei***
7	Inflorescence subglabrous	***H. cavaleriei***
–	Inflorescence densely glandular puberulent	**8**
8	Stems ascending, flexuous	***H. flexuosa***
–	Stems erect, straight	***H. mairei***


## Supplementary Material

XML Treatment for Hanceola suffruticosa
